# Replacement of endoprosthetic implants within a two years follow-up period: a statutory health insurance routine data analysis

**DOI:** 10.1186/1471-2474-13-223

**Published:** 2012-11-16

**Authors:** Roland Linder, Hardy Müller, Brigitte Grenz-Farenholtz, Caroline Wagner, Martin Stockheim, Frank Verheyen

**Affiliations:** 1 Wissenschaftliches Institut der TK für Nutzen und Effizienz im Gesundheitswesen (WINEG) (Scientific Institute of the Techniker Krankenkasse (TK) for Benefit and Efficiency in Health Care), Bramfelder Straße 140, Hamburg, 22305, Germany; 2 St.-Marien-Hospital Borken, Clinic for Orthopaedics and Trauma Surgery, Borken, Germany

**Keywords:** Endoprostheses, Hip joint, Knee joint, Routine data analysis, Arthroplasty register

## Abstract

**Background:**

The statutory health insurance system embodies a large amount of data on the treatments of their members. Depending on joint, prosthesis type, patient activity and comorbidity, knee and hip replacements can last up to 20 years. Based on statutory health insurance data the main object of this analysis was to investigate how high the early revision rate of replacements actually is.

**Methods:**

The number of replacements in the years 2005 and 2006 has been extracted from the TK database for hip (OPS-Code 5-820, n = 20,875), knee (OPS 5-822, n = 13,466), upper limbs (OPS 5-824, n = 901), and lower limbs (OPS 5-826) replacements. This data has then been related to each consecutive operation (i. e. change or excision of joint endoprosthesis) over a joint-specific observation period of two years.

**Results:**

In 3.7% of the cases joint replacements stood for less than 2 years (hip 3.5%, knee 3.8%, upper limbs 6.5%, and lower limbs 5.5%). There is a significantly positive correlation between the treatment data of the hospitals and the outcome as to low rates of reoperations at early stages. The main reason for short lifetime (76 - 81%) is mechanical failure.

**Conclusion:**

The percentage of joint endoprostheses with significantly short lifetimes has been unexpectedly high. The de facto lifetimes of joint endoprostheses thus often do not match the manufacturers’ information. The authors strongly support the idea of a national endoprosthesis register as such a register could give detailed information on

firstly whether these deficits are due to material defects, osteolysis or dislocation and

secondly which products are mainly affected.

## Background

Manufacturers specify that the lifetimes of joint endoprostheses, i. e. the time between first operation and the first reoperation, is 15 years and above. However, joint endoprostheses often fail before that. Among others, the two main reasons are material defects or faulty constructions as was frequently published in the press with high circulation last year [1-3]. In March 2010, a hospital had to change 125 defect hip prostheses because cuttings of a special type of hip endoprostheses detached [4].

A change of joint endoprosthesis is indicated in case of aseptic or septic loosening, periprosthetic fracture caused by a trauma or material defects. Progressive attrition in the joint parts not yet replaced (e.g. partial replacement of knee joints) or major functional impairment after joint replacement may also indicate a change. For a detailed list of relevant complications see Saleh et al. 2002 [5]. Recently Anand et al. published a survey on the market penetration and performance of newly introduced hip and knee prostheses. Between 2003 and 2007 266 new hip or knee endoprostheses were introduced into the Australian market. About one third of the new knee prostheses and a fifth of the new hip prostheses came to use in more than 100 procedures and were subject to evaluation. Roughly a third of the new prostheses showed higher revision rates than well established models [6].

According to the figures published in the BQS quality reports^a^ in 2004 a total number of 137,858 primary hip replacements and 17,696 revision hip arthroplasties were carried out [7]. In 2008 these figures rose to 156,803 primary hip replacements and 22,628 revision hip arthroplasties [8]. The number of primary hip arthroplasties increased by 13.7%, whereas the revision hip arthroplasties rose by double (27.9%), see Tables 1 and 2.

**Table 1:** **Development of primary total hip arthroplasty and hip revision surgery 2004-2008**[7,8]

**Year 2004 (n)**		**Year 2008 (n)**		**Changes from 2004 to 2008 (%)**	
**Primary total hip replacements**	**Joint changes**	**Primary total hip replacements**	**Joint changes**	**Primary total hip replacements**	**Joint changes**
137,858	17,696	156,803	22,628	+13.7	+27.9

**Table 2:** **Development of primary total knee arthroplasty and knee revision surgery 2004-2008**[7,8]

**Year 2004 (n)**		**Year 2008 (n)**		**Changes from 2004 to 2008 (%)**	
**Primary total knee replacements**	**Joint changes**	**Primary total knee replacements**	**Joint changes**	**Primary total knee replacements**	**Joint changes**
110,349	7,238	145,996	10,376	+32.6	+43.4

A precise analysis of these findings is impossible, due to the limitations of the data available. Neither the data transferred to the statutory health insurance funds nor the data collected by the BQS contains the needed information about the precise type of prosthesis. The follow-up data is also limited: the observation horizon either ends with hospital demission of the patient (data of the BQS) or when the patient changes the statutory health insurance fund (the statutory health insurance data).

Material defects may cause serial defects which can result in a large number of failures concerning one product. A mechanical defect - e. g. a founding fault - can lead to the break of a large number of implants. For example a specific series of hip joint components of an US manufacturer loosened early because of lubricant residues on the surface of the components in 2000. According to press reports 5,500 to 6,000 prostheses of the withdrawn surface replacing prosthesis “ASR” of the DePuy Company have been implanted [9,10]. Exact figures are not available, since a central national register for joint replacement does not exist yet. Therefore currently the only way to investigate the specific research questions is by using intersectoral data of a large nationwide statutory health insurance fund covering several millions of insurants.

## Methods

The data analysed, consists of patient specific data which have to be transferred from the hospital to the health insurance funds for every inpatient treatment. The data comprise different items, i.e. age, sex, date of hospital admittance, diagnosis, treatment, reimbursement code etc. as well as identification numbers which allows for individual patient follow-up. Initially data is collected for payment procedures. Since all data have been anonymised and since exclusively aggregated statistics have been generated, there is no need to ask for an ethic approval. The focus was set on the diagnosis and treatment codes (ICD10-GM and OPS), of patients who had continuously been insured with the TK from 2005-01-01 to 2009-12-31. The OPS is the German Procedure Classification published by the German Institute for Medical Documentation and Information (DIMDI; “Deutsches Institut für Medizinische Dokumentation und Information”). The ICD10-GM, the German Modification of the International Classification of Diseases and Related Health Disorders, used are from the catalogue versions of the year in question. The focus was set on the three-figure OPS 5-82 (endoprosthetic replacement of joint and bone) in comparison with the four-figure OPS for implantation of joint endoprostheses 5-820 (hip), 5-822 (knee), 5-824 (joints of the upper limbs: shoulder, elbow, wrist, and finger arthroplasty), and 5-826 (joints of the lower limbs: ankle, forefoot, and toe arthroplasty) as well as the corresponding revisions which lead to a replacement or removal of joint endoprostheses (5-821, 5-823, 5-825, and 5-827). Revisions which left the implant unchanged were not taken into consideration (5-821.0, 5-823.0, 5-825.0, and 5-827.0). The following ICD-codes were evaluated for the purpose of causal research: figures T 84.0 and T 84.4 combined as “mechanical complications”, figures T 84.0 and T 84.7 combined as “infection/inflammation”, as well as figures T 84.8 (“other complications”) and T 84.9 (“complications not exactly specified”).

Joint endoprostheses implantations between 2005-01-01 and 2007-12-31 were identified under the assumption of being primary implantations. The only inclusion criteria were the aforementioned OPS-codes. Exclusion criteria have not been applied. Based on the relevant operation date the corresponding patient was being monitored over a period of two years. New operations in terms of repeated changes of implants or new implantations after removal of the former prosthesis were not taken into consideration. The observation period of two years for short-term reoperations equals the period which is the indicator of “short-term complications”. This indicator is included as national quality indicator in the report “Quality and Efficiency in Swedish Health Care. Regional Comparisons 2007” of the Swedish Association of Local Authorities and Regions, SALAR, and Swedish National Board of Health and Welfare, SoS [11,12].

The two-tailed Mann–Whitney-*U*-Test was applied to the comparative examination between patients with and without short-term reoperations, between hospitals with and without a defined minimum number of operations per year, and hospitals with and without an integrated health care contract with the statutory health insurance fund Techniker Krankenkasse, furthermore referred to as TK, respectively. Regarding hospital procedure volumes a threshold of 100 operations annually has been chosen because this threshold has already been used by Katz et al. in their prominent study on the association between procedure volumes and outcomes [13]. Subsequently this has been adopted by the German Society for Orthopaedics and Orthopaedic Surgery and the German company ClarCert in Neu-Ulm who jointly developed a concept for attesting centres specialised on endoprostheses operations. From an expert point of view a threshold of 100 is feasible and scientifically adequate.

The significance level was set at 0.05. Finally 4-figure and 5-figure OPS have been listed with the aggregation of (normally 6-figure) OPS-figures without indicating a possible more detailed specification.

## Results

5,027,709 individuals were insured at TK during the observation period from 2005-01-01 until 2007-12-31. Within the observation period the population received a total of 20,875 hip replacements, moreover 13,466 total knee replacements, 901 upper limb endoprostheses, 128 lower limb endoprostheses (for demographics see Table 3). The percentage of short-term reoperations of hip endoprostheses amounted to 3.47% (knee 3.81%, upper limb 6.55%, and lower limb 5.47%) during the two years of postoperative observation.

**Table 3:** Distribution on sex and age

**Location**	**Sex**	**Median age**	**Age range**
Total hip replacement	m	68	16 - 100
(n = 20,875)	f	69	15 - 104
Total knee replacement	m	69	15 - 97
(n = 13,466)	f	69	12 - 95
Upper limb endoprosthesis	m	66	14 - 91
(n = 901)	f	70	23 - 96
Lower limb endoprosthesis	m	64	27 - 82
(n = 128)	f	61	28 - 79

Over the whole period of two years the percentage of mechanical complications regarding all four joint localisations amounted from 76% to 81% as to the total number of reoperations, the rate of infections and inflammations amounted from 18% to 19%. A significant difference between the joint localisations is not evident.

Concerning hips, the index surgery most often leading to revision are 5-820.5 (acetabular revision cup/cage systems 29.2%), 5-820.7 (snap-fit acetabular cup12.5%), and 5-820.3 (femoral head prosthesis 5.7%).

Regarding knees, the index surgery most frequently effecting revision are 5-822.0 (uni-compartmental knee 9.0%), 5-822.x (other endoprostheses 7.5%), and 5-822.b (endoprostheses with extended flexor capability and patella supplementation 6.3%). The OPS-code 5-822.b was first listed in the OPS version of 2007.

**Figure 1:**
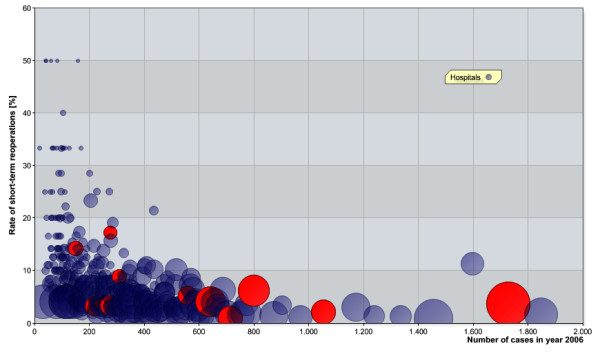
**Scatter plot: Each circle stands for a hospital.** Its area correlates with the number of hip joint endoprostheses implantations of the persons insured at TK. The abscissa shows the annual number of cases according to BQS [7]. Those hospitals with integrated health care contracts with TK for endoprosthetic care are marked in red.

**Figure 2:**
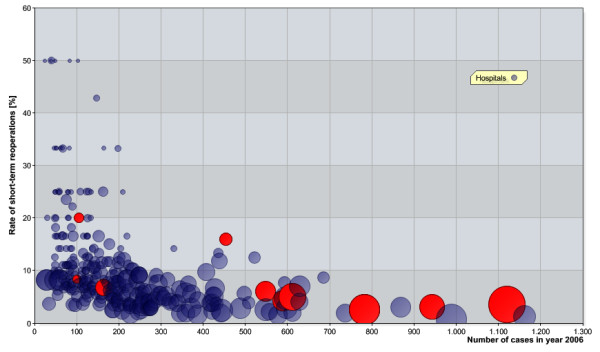
**Scatter plot as in Figure**1**but for knee endoprosthetic joint implantations.**

The relation between the annual number of cases of joint replacement surgery per institution and the rate of short-term reoperations (see Figures 1 and 2, as well as Tables 4 and 5) shows a negative correlation for a level of 100. There is a highly significant difference between the rates of reoperations at clinics. The latter perform more than 100 primary implantations (*p* < 0.001) of both hips and knees. It is important to emphasise that rates of short-term reoperations listed in Tables 4 and 5 are higher than the values given above. This is probably a consequence of a couple of large hospitals which are of outstanding quality. For instance, the top three hospitals in terms of procedure volumes yield 1.9% as a rate of short-term reoperations regarding hip joints (knee endoprostheses 2.8%).

**Table 4:** Differences between hospitals with low and high procedure volumes (threshold = 100 operations annually)

**Number of hip joint endoprostheses implantations of the persons insured at TK**	**Rate of short-term reoperations in hospitals with < 100 operations p.a. (%, median)**	**Rate of short-term reoperations in hospitals with > = 100 operations p.a. (%, median)**	***p *value**
< 10	20.0 (44 hospitals)	14.3 (32 hospitals)	0.070
10 - 19	10.0 (19 hospitals)	7.7 (72 hospitals)	0.111
> = 20	4.76 (5 hospitals)	4.2 (211 hospitals)	0.304
Total	14.3 (68 hospitals)	5.7 (315 hospitals)	< 0.001

Number of hip joint endoprostheses of the persons insured at TK serves as an indicator for confidence.

**Table 5:** Differences between hospitals with low and high procedure volumes (threshold = 100 operations annually)

**Number of knee joint endoprostheses implantations of the persons insured at TK**	**Rate of short-term reoperations in hospitals with < 100 operations p.a. (%, median)**	**Rate of short-term reoperations in hospitals with > = 100 operations p.a. (%, median)**	***p *value**
< 10	22.2 (47 hospitals)	16.7 (24 hospitals)	0.229
10 - 19	9.1 (31 hospitals)	8.3 (54 hospitals)	0.481
> = 20	8.2 (10 hospitals)	4.8 (136 hospitals)	0.058
Total	14.3 (88 hospitals)	6.6 (214 hospitals)	< 0.001

Number of knee joint endoprostheses of the persons insured at TK serves as an indicator for confidence.

The patients’ age in facilities with a proportion of short-term reoperations ≥ 20% was compared to the patients’ age in other facilities with better outcome to ensure that patients are considered from facilities with various complication rates. For instance, older patients (who are thus more often ill) are treated in smaller institutions close to their homes. Significant differences as to joints of hip or knee are not apparent (*p* = 0.990 and *p* = 0.766, respectively).

Considering short-term complications as a quality indicator like the Swedes do (as mentioned before), hospitals with integrated health care contracts with TK for endoprosthetic care have by tendency a high quality treatment as to the rate of short-term reoperations (hip: *p* = 0.121, knee: *p* = 0.132), see Figures 1 and 2.

Matrixes can be plotted for the different joints on the basis of statutory health insurance routine data (not illustrated). They relate the OPS-codes of initial operations to the OPS-codes of early reoperations. Possible additional dimensions of such a matrix are: the definition of the primary disease at time of implantation (osteoarthrosis, rheumatoid arthritis, fractures close to joint), the distinction between cemented and non-cemented implants (6^th^ position of OPS-code), or the type of complication which led to the reoperation (mechanical problems or infections). For instance, total hip replacement (OPS 5-820.0) with an early subsequent removal of the endoprosthesis (OPS 5-821.7) showed to be three times more induced by infections (ICD T84.5 and T84.7) than by mechanical complications (ICD T84.0 and T84.4). The age distribution of patients with short-term reoperations was compared with that of patients without short-term reoperations to check whether implantations in older patients were less carefully executed than in younger ones. Significant differences did not arise with hip endoprostheses (patients with short-term reoperation: median age = 67 years, patients without short-term reoperation: = 67 years, *p* = 0.533). A significant difference of *p* < 0.001 occurred, though, for knee endoprostheses. The median age of 67 for patients with short-term reoperation is one year below the median age of the reference group (median age = 68 years).

## Discussion

Due to the increase in musculoskeletal disorders of an aging population the World Health Organization (WHO) and the United Nations (UN) have declared the years 2000 to 2010 as to be the “Bone and Joint Decade”. In the beginning of the next decade, the booming supply of endoprostheses will still be a major issue. The large number [14] as well as the combination varieties of new prostheses modules urgently requires a quality ensuring joint replacement register.

### Result Discussion

Comparing accounting data and statutory health insurance data with registry data has limitations. The patient relevant outcome (time between primary implantation and revision surgery), however, can be extracted from both data sets and it is possible to compare these values. Comparing the present findings with data from the Swedish Hip Arthroplasty Register [12], one finds significantly smaller early revision rates than in the population of TK insurants. In this context the average early revision rate between 2005 and 2008 is 1.6% with a county council spread of 0.6% to 2.3%. Coincidently, a national comparison with the data of AOK (Local Health Care Fund in Germany) shows similar complication rates within an observation period of one year after hip endoprosthesis with coxarthrosis (2.8%), hip endoprosthesis with hip fracture (3.8%), and after knee endoprosthesis (1.7%) [15].

Most prostheses loosenings are due to aseptic loosenings according to the results published by BQS [8]. This can be attributed to local inflammatory reactions of tissue to micro wear particles of the prosthesis material or bony transformation as reaction to a changed mechanical situation. This result has also been internationally reported (Sweden [16], Australia [17], England/Wales [18]).

Regarding possible differences in age distribution of patients with and without short-term reoperation, it cannot be concluded that the reason is increased exposure of younger patients’ endoprostheses or rather the lack of a possible surgery due to medical reasons or the less frequent wish of older patients to be reoperated.

Considering the top three index surgeries most often leading to revision, knee prosthesis designed for higher flexion (high-flex knees, OPS 5-822.b) is of special interest: The findings presented support the findings of Hamilton et al. [19] that high flex knee prosthesis shows a higher risk of early loosening. Endres [20] has shown that patients with high-flex knee prosthesis achieve no better range of motion than patients with usual knee prosthesis. It can be concluded that the patient related benefit of high-flex knee prosthesis is at least unclear, presumably not existent, but exposes the patient to a higher risk of earlier revision.

The Institute for Quality and Efficiency in Statutory Health Insurance, furthermore referred to as IQWiG (“Institut für Qualität und Wirtschaftlichkeit im Gesundheitswesen”) has carried out an investigation to level calculation and identified a U-shaped course between the number of cases and the risk for complications considering knee endoprostheses [21]. Instead of the frequency of short-term reoperations the risk of a restricted postoperative joint mobility and postoperative wound infection has been analysed. These IQWiG results do not contradict the results presented in this publication. However, it is not the aim of the authors to foster the challenging discussion on minimum quantities [22,23]. Instead, the authors would like to put emphasis on quality indicators in order to evaluate the success of treatment. The former are currently gaining importance in the context of selective contracts for integrated care, gatekeeper centred health care and disease managements programmes [24].

### Limitations and strengths

The Techniker Krankenkasse has more than 8 million insurants representing approximately 10 percent of the inhabitants of the federal Republic of Germany. At TK, the sample analysed is a full sample and therefore representative for TK insurants. However, characteristics of the specific characteristics of TK insurants may differ from the entire German population *e.g*. regarding the socio-economic status and thereby the BMI that on his part may influence the rate of short-term reoperations. Unfortunately, with exception of somewhat additional documentation like for participants in disease management programs, there is no possibility to control confounders. Therefore it will be not allowed to extrapolate the results to the entire population.

The authors would have preferred to apply other quality indicators such as the “ten-year prosthesis survival” used by SALAR and SOS [11] but the localisation characters “R” (right sided), “L” (left sided), or “B” (both sides) relevant for the implant classification has only been documented since 2005-01-01.

The data on both hip and knee endoprostheses comprised more than 34,000 total joint replacements including follow-up and was thus comprehensible enough. Between 2005 and 2007 endoprostheses for shoulder, ankle, or elbow were so seldom implanted that reliable data to the joints of upper and lower limbs do not yet exist.

The assumption is that the de facto percentage of short-term complications will be higher than reported, since inoperable patients, the deceased and those who change their statutory health insurance fund have not been considered. Please note that the reported figure for primary implantations cannot be used for epidemiological information: On the one hand, the TK insurants differ from the total population of insurants in German statutory health insurance funds. On the other hand the complex primary implantations which are separately to encode (OPS 5-829.a) were just not considered as such as the use of tumour endoprostheses (OPS 5-829.c) or hypoallergenic prostheses (OPS 5-829.e). The OPS-catalogue has been adjusted several times during the observation period of primary implantations. The OPS 5-820.6 (femoral head) has been abandoned with the OPS-catalogue 2007 introducing the OPS 5-820.8 (hip resurfacing). The OPS 5-820.9 (neck-conserving femoral head prosthesis) used in the current OPS-catalogue 2010 was not evaluated since it has only been introduced with the OPS-catalogue 2008.

### Outlook

In addition to reoperations it is without doubt necessary to investigate re-reoperations, thus following the Swedish example [12]. Moreover it would be enriching not only to focus on complications, but to consider all others medical services demanded in the context of the treatment. The data of statutory health insurance funds for example offers valuable data on post-hospital rehabilitation [25] or ambulatory care, physiotherapy and further medication. It also includes the ICD-codes not only of the primary diseases (e.g. coxarthrosis or rheumatoid arthritis) but also of the relevant comorbidities. In contrast to the options of the aggregated short-term information of hospitals transferred to the BQS the data of the statutory health insurance funds allows morbidity-orientated matching and benchmarking. Furthermore reoperation data can also be referred to the ICD-codes for injuries or periprosthetic fractures (ICD S72, S82). An overall analysis of joint replacement and all other related medical services would generate findings of the complete picture. Evaluation of statutory health insurance data will always have limitations (e.g. missing data on implants and operation techniques, the subjectiveness of patient evaluation or deficits in documentation). Nevertheless this data will deliver valuable, new findings on joint replacements and further research questions, especially when connected to register data.

### Arthroplasty register

Hardly anybody doubts the medical necessity of a high quality arthroplasty register [26,27]. It is the basis for callbacks as known from the car industry where it is possible to unambiguously allocate vehicles to owners. An arthroplasty register will lead to valid revision rates which are four to fourteen times underestimated in clinical literature [28]. And last but not least an arthroplasty register is highly interesting regarding economic aspects: The German Society for Orthopaedics and Orthopaedic Surgery, (DGOOC, “Deutsche Gesellschaft für Orthopädie und Orthopädische Chirurgie”) reckons savings of 45 million Euro in the third year of the register. In contrast, additional costs of the register will be distinctly less [29]. A joint replacement register must necessarily have an implant to patient allocation and could usefully be supplemented by selected statutory health insurance routine data.

## Conclusions

Our analysis shows quality deficits in joint replacements. The current state of joint replacement in Germany contradicts the German social legislation which calls for a highly qualified service according to the current state of scientific and medical knowledge. A national arthroplasty register could help to detect problems at a very early stage as it monitors the service providers and medical products under daily conditions. This has already been proven in other countries. It is time to give German patients and physicians equal opportunities, better treatment options and avoid avoidable damage.

## Endnote

^a^BQS (“Institut für Qualität und Patientensicherheit - Bundesgeschäftsstelle Qualitätssicherung GmbH”) is a non-profit organisation which was commissioned to be in charge of the quality assurance of the German hospitals from 2001 to 2009. BQS was commissioned by the umbrella organisation of the statutory health insurance funds (“Spitzenverbände der gesetzlichen Krankenkassen”) together with the Central Association of Private Health Insurance (“Verband der privaten Krankenversicherung”), the German Hospital Federation (“Deutsche Krankenhausgesellschaft”), the German Medical Association (“Bundesärztekammer”) and the German Care Council (“Deutscher Pflegerat”). As a result of a tendering procedure the Institute for Applied Quality Improvement and Research in Health Care (“AQUA-Insitut für angewandte Qualitätsförderung und Forschung im Gesundheitswesen GmbH”) has undertaken this task since 2010.

## Competing interests

The mission of the Scientific Institute of the TK for Benefit and Efficiency in Health Care (Wissenschaftliches Institut der TK für Nutzen und Effizienz im Gesundheitswesen, WINEG) is to investigate the value of innovations and new programmatic approaches within the statutory health insurance framework. Five of six authors declare that because they belong to the Techniker Krankenkasse, a potential competing interest exists according to the guidelines of the International Committee of Medical Journal Editors.

## Authors’ contributions

RL, HM, and FV participated in the design of the study; BGF and MS did the literature review; RL performed the statistical analysis; RL, MS, and CW participated in drafting the manuscript also translating paragraphs from German language into English language. All authors read and approved the final manuscript.

## Authors’ information

Co-authors: Hardy Müller, M.A., Brigitte Grenz-Farenholtz, Ass. jur., Dr. rer. pol. Caroline Wagner, MSc, Martin Stockheim and Dr. rer. nat. Frank Verheyen.

## Pre-publication history

The pre-publication history for this paper can be accessed here:

http://www.biomedcentral.com/1471-2474/13/223/prepub
